# Tetra­kis(μ_2_-cyanido-κ^2^ *C*:*N*)dicyanido­tetra­kis­[tris­(2-amino­eth­yl)amine-κ^3^ *N*,*N*′,*N*′′,*N*′′′]tetra­copper(II)iron(II) bis[pentacyanidonitrosoferrate(II)] hexahydrate

**DOI:** 10.1107/S1600536812038251

**Published:** 2012-09-15

**Authors:** Olesia V. Kozachuk, Julia A. Rusanova, Oksana V. Nesterova, Roman Zubatyuk, Oleg V. Shishkin

**Affiliations:** a Department of Inorganic Chemistry, Taras Shevchenko National University of Kyiv, 64 Volodymyrska St, Kyiv 01601, Ukraine; b Department of Inorganic Chemistry II, Ruhr-University Bochum, Universitatstrasse 150, 44801 Bochum, Germany; c Division of Functional Materials Chemistry, SSI "Institute for Single Crystals", National Academy of Science of Ukraine, 60 Lenina Ave., Kharkiv 61001, Ukraine; d Department of Inorganic Chemistry, V. N. Karazin National University, 4 Svobody Sq, Kharkiv 61077, Ukraine

## Abstract

The asymmetric unit of the title complex, [Cu_4_Fe(CN)_6_(C_6_H_18_N_4_)_4_][Fe(CN)_5_(NO)]_2_·6H_2_O, comprises a complex [{Cu(tren)CN}_4_Fe(CN)_2_]^4+^ [tren is tris­(2-amino­eth­yl)amine] cation, which exhibits -1 symmetry with the terminal cyanide ligands oriented *trans* to each other, and two [Fe(CN)_5_(NO)]^2−^ nitroprussiate counter-anions. In the crystal, N—H⋯N hydrogen-bonding inter­actions are observed between H atoms on the primary amine groups of the tren ligand and the terminal cyanide groups of the nitro­prussiate counter-ions. The N atom in the terminal CN ligand of the cation is equally disordered over two positions. The structure also contains disordered lattice water mol­ecules. Their contribution was elimin­ated from the refinement using the procedure described by van der Sluis & Spek (1990 ▶).

## Related literature

For background to direct synthesis, see: Nesterov *et al.* (2004 ▶, 2006 ▶); Nesterova *et al.* (2004 ▶); Pryma *et al.* (2003 ▶)Vinogradova *et al.* (2002 ▶); Makhankova *et al.* (2002 ▶); Babich *et al.* (1996 ▶). For the structures of related complexes, see: El Fallah *et al.* (1996 ▶); Lu *et al.* (1997 ▶); Zou *et al.* (1997 ▶); Parker *et al.* (2001 ▶) The contribution from disordered water mol­ecules was eliminated using the *OLEX2* interface; for background, see: van der Sluis & Spek (1990 ▶).
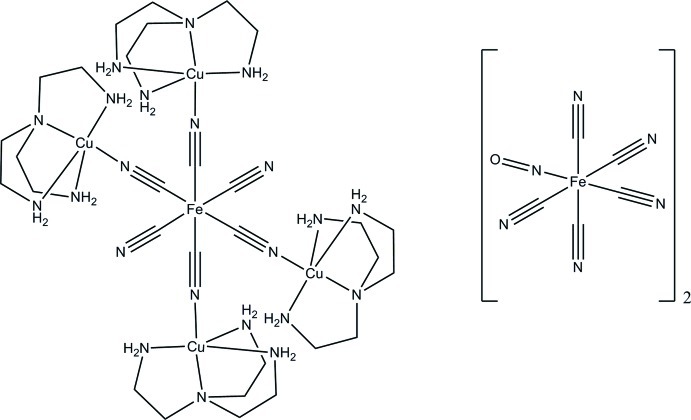


## Experimental

### 

#### Crystal data

[Cu_4_Fe(CN)_6_(C_6_H_18_N_4_)_4_][Fe(CN)_5_(NO)]_2_·6H_2_O
*M* *_r_* = 1591.16
Triclinic, 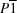

*a* = 7.9270 (2) Å
*b* = 14.9656 (4) Å
*c* = 17.5565 (4) Å
α = 114.879 (3)°
β = 94.021 (2)°
γ = 98.909 (2)°
*V* = 1845.30 (8) Å^3^
*Z* = 1
Mo *K*α radiation
μ = 7.76 mm^−1^
*T* = 100 K
0.24 × 0.17 × 0.06 mm

#### Data collection

Agilent Xcalibur Sapphire3 diffractometer
Absorption correction: analytical [*CrysAlis PRO* (Agilent, 2011 ▶), based on expressions derived by Clark & Reid (1995 ▶)] *T*_min_ = 0.752, *T*_max_ = 0.909
32856 measured reflections
9691 independent reflections
6403 reflections with *I* > 2σ(*I*)
*R*_int_ = 0.040

#### Refinement

*R*[*F* ^2^ > 2σ(*F* ^2^)] = 0.051
*wR*(*F* ^2^) = 0.138
*S* = 1.05
9691 reflections
385 parameters
H-atom parameters constrained
Δρ_max_ = 1.49 e Å^−3^
Δρ_min_ = −0.68 e Å^−3^

### 

Data collection: *CrysAlis PRO* (Agilent, 2011 ▶); cell refinement: *CrysAlis PRO*; data reduction: *CrysAlis PRO*; program(s) used to solve structure: *SHELXS97* (Sheldrick, 2008 ▶); program(s) used to refine structure: *SHELXL97* (Sheldrick, 2008 ▶); molecular graphics: *OLEX2* (Dolomanov *et al.*, 2009 ▶); software used to prepare material for publication: *publCIF* (Westrip, 2010 ▶).

## Supplementary Material

Crystal structure: contains datablock(s) global, I. DOI: 10.1107/S1600536812038251/hg5241sup1.cif

Structure factors: contains datablock(s) I. DOI: 10.1107/S1600536812038251/hg5241Isup2.hkl

Supplementary material file. DOI: 10.1107/S1600536812038251/hg5241Isup3.cdx

Additional supplementary materials:  crystallographic information; 3D view; checkCIF report

## Figures and Tables

**Table 1:** Hydrogen-bond geometry (Å, °)

*D*—H⋯*A*	*D*—H	H⋯*A*	*D*⋯*A*	*D*—H⋯*A*
N4—H4*A*⋯N15^i^	0.92	2.20	3.020 (4)	149
N4—H4*B*⋯N16^ii^	0.92	2.37	3.248 (4)	159
N5—H5*A*⋯N7*A* ^iii^	0.92	2.09	2.979 (9)	162
N5—H5*A*⋯N7*B* ^iii^	0.92	2.41	3.267 (9)	154
N6—H6*A*⋯N17^ii^	0.92	2.43	3.242 (5)	147
N10—H10*A*⋯N17^iv^	0.92	2.29	3.060 (5)	141
N10—H10*B*⋯N16^v^	0.92	2.27	3.151 (5)	160

Symmetry codes: (i) 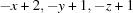; (ii) 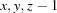; (iii) 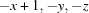; (iv) 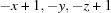; (v) 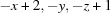.

**Fig. 1.**
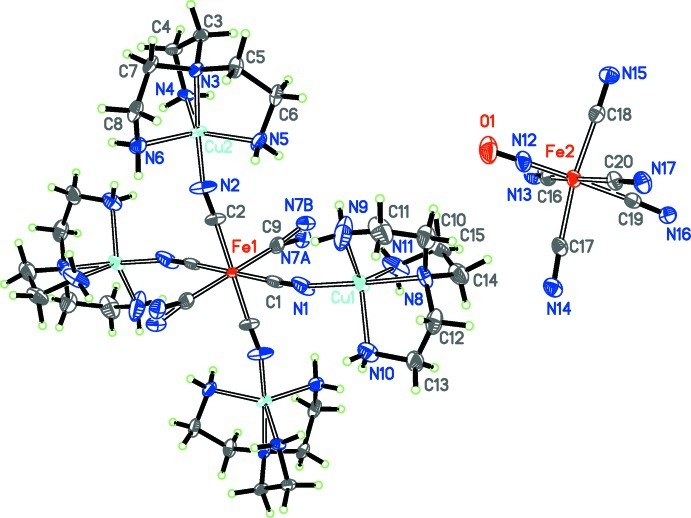
Structure of the title compound, with displacement ellipsoids drawn at the 30% probability level for non-H atoms. Unlabelled atoms are generated by the application of the inversion centre.

## supplementary crystallographic information

### Comment

As it was shown in our previuous publication direct synthesis is an efficient method to obtained novel homo- and heterometallic complexes (Nesterov *et al.*, 2004, 2006; Nesterova *et al.*, 2004; Pryma *et al.*, 2003; Vinogradova *et al.*, 2002; Makhankova *et al.*, 2002); Babich *et al.*, 1996). In this paper we present a novel Cu/Fe heterometallic *ionic* complex which has been synthesized using zerovalent copper, Sodium nitroprusside and tris(2-aminoethyl)-amine as starting materials.

The asymmetric unit contains two iron ions. One of them which is connected to copper ions by bridging cyanide groups is localized at the special equivalent position *(O,O,O)* (Fig. 1). The Cu—Fe separations range between 4.9044 (5) and 4.9403 (5) Å. Each Cu located in the center of a distorted trigonal bipyramid formed by four nitrogen atoms of the *tren* ligand and one nitrogen atom of cyanide groups. The Cu—N distances range between 2.048 (2) and 2.103 (3) Å for Cu—Ntren) and between 1.932 (3) and 1.947 (3) Å, for Cu—NCN). The Fe1—C distances range from 1.886 (3) to 1.912 (4) Å, whereas, as expected, the Fe—C—N bond angles only vary in the small range between 176.1 (4)° and 176.5 (4)° (not taking into account disordered CN ligand). The Cu—N—C bond angles, on the other hand, deviate significantly from linearity and lie between 163.4 (4) and 165.0 (4)°. All bond distances and angles are comparable to the corresponding distances in closely related compounds (El Fallah *et al.* (1996); Lu *et al.* (1997); Zou *et al.* (1997); Parker *et al.* (2001)).

### Experimental

The title compound was prepared by direct synthesis mixing of the zerovalent copper powder (0,079 g, 1,25 mmol), NH_4_NCS (0,096 g, 1,26 mmol), Na_2_[Fe(CN)_5_(NO)].2H_2_O (0,188 g, 0,63 mmol), tris(2-aminoethyl)-amine (0,19 ml, 1,27 mmol), methanol (30 ml) were heated to 323–333 K and stirred magnetically for 130 min. Resulted mixture was filtered off and transparent brown solution was allowed to stand at room temperature. Dark brown square plate crystals suitable for X-ray analysis precipitated within two months by adding of 5 ml of diethyl ether.They were collected by filter-suction, washed with dry Pr^í^OH and finally dried in *vacuo* at room temperature (yield: 0.14 g, 30%)

### Refinement

All H atoms were refined using rigid model with *U*_iso_ = 1.2*U*_eq_ of the carrier atom. The N atom in one of the CN ligands is disordered over two positions with equal occupancy. Despite of the fact that some other atoms show high *U*_eq_ or prolate U_aniso_, F_obs_ map indicates single electron density peak for each atom. Thus, no disorder was introduced in the model. Structure contains disorderes water molecules. Solvent contribution was eliminated using procedure described by van der Sluis and Spek (1990). Integrated number of solvent electrons per cell is 39.9, which corresponds to 4 water molecules.

### Crystal data

**Table:**

[Cu_4_Fe(CN)_6_(C_6_H_18_N_4_)_4_][Fe(CN)_5_(NO)]_2_·6H_2_O	*Z* = 1
*M**_r_* = 1591.16	*F*(000) = 820
Triclinic, *P*1	*D*_x_ = 1.432 Mg m^−^^3^
Hall symbol: -P 1	Mo *K*α radiation, λ = 0.7107 Å
*a* = 7.9270 (2) Å	Cell parameters from 6786 reflections
*b* = 14.9656 (4) Å	θ = 2.9–30.3°
*c* = 17.5565 (4) Å	µ = 1.77 mm^−^^1^
α = 114.879 (3)°	*T* = 100 K
β = 94.021 (2)°	Block, brown
γ = 98.909 (2)°	0.24 × 0.17 × 0.06 mm
*V* = 1845.30 (8) Å^3^	

### Data collection

**Table:**

Agilent Xcalibur Sapphire3 diffractometer	9691 independent reflections
Radiation source: Enhance (Mo) X-ray Source	6403 reflections with *I* > 2σ(*I*)
Graphite monochromator	*R*_int_ = 0.040
Detector resolution: 16.1827 pixels mm^-1^	θ_max_ = 30.3°, θ_min_ = 2.9°
ω scans	*h* = −11→10
Absorption correction: analytical [*CrysAlis PRO* (Agilent, 2011), based on expressions derived by Clark & Reid (1995)]	*k* = −20→21
*T*_min_ = 0.752, *T*_max_ = 0.909	*l* = −22→24
32856 measured reflections	

### Refinement

**Table:**

Refinement on *F*^2^	Primary atom site location: structure-invariant direct methods
Least-squares matrix: full	Secondary atom site location: difference Fourier map
*R*[*F*^2^ > 2σ(*F*^2^)] = 0.051	Hydrogen site location: inferred from neighbouring sites
*wR*(*F*^2^) = 0.138	H-atom parameters constrained
*S* = 1.05	*w* = 1/[σ^2^(*F*_o_^2^) + (0.069*P*)^2^ + 0.5531*P*] where *P* = (*F*_o_^2^ + 2*F*_c_^2^)/3
9691 reflections	(Δ/σ)_max_ < 0.001
385 parameters	Δρ_max_ = 1.49 e Å^−^^3^
0 restraints	Δρ_min_ = −0.68 e Å^−^^3^

### Special details

**Table:**

Experimental. Absorption correction: CrysAlisPro, Agilent Technologies, Version 1.171.35.19 (release 27-10-2011 CrysAlis171 .NET) (compiled Oct 27 2011,15:02:11) Analytical numeric absorption correction using a multifaceted crystal model based on expressions derived by R.C. Clark & J.S. Reid. (Clark, R. C. & Reid, J. S. (1995). Acta Cryst. A51, 887-897)
Geometry. All e.s.d.'s (except the e.s.d. in the dihedral angle between two l.s. planes) are estimated using the full covariance matrix. The cell e.s.d.'s are taken into account individually in the estimation of e.s.d.'s in distances, angles and torsion angles; correlations between e.s.d.'s in cell parameters are only used when they are defined by crystal symmetry. An approximate (isotropic) treatment of cell e.s.d.'s is used for estimating e.s.d.'s involving l.s. planes.
Refinement. Refinement of *F*^2^ against ALL reflections. The weighted *R*-factor *wR* and goodness of fit *S* are based on *F*^2^, conventional *R*-factors *R* are based on *F*, with *F* set to zero for negative *F*^2^. The threshold expression of *F*^2^ > σ(*F*^2^) is used only for calculating *R*-factors(gt) *etc*. and is not relevant to the choice of reflections for refinement. *R*-factors based on *F*^2^ are statistically about twice as large as those based on *F*, and *R*- factors based on ALL data will be even larger.

### Fractional atomic coordinates and isotropic or equivalent isotropic displacement parameters (Å^2^)

**Table:**

	*x*	*y*	*z*	*U*_iso_*/*U*_eq_	Occ. (<1)
Cu1	0.83068 (6)	0.02273 (3)	0.26993 (3)	0.04262 (13)	
Cu2	0.68711 (5)	0.27612 (3)	0.05083 (3)	0.03257 (12)	
Fe1	1.0000	0.0000	0.0000	0.02668 (15)	
Fe2	0.78985 (6)	0.31396 (4)	0.70855 (3)	0.03349 (13)	
O1	0.5468 (4)	0.3216 (3)	0.5890 (2)	0.0744 (10)	
N1	0.8551 (6)	−0.0065 (3)	0.1535 (2)	0.0769 (14)	
N2	0.8147 (5)	0.1677 (2)	0.0138 (3)	0.0627 (11)	
N3	0.5695 (3)	0.3970 (2)	0.09740 (16)	0.0278 (6)	
N4	0.8633 (3)	0.36876 (18)	0.02108 (17)	0.0292 (6)	
H4A	0.9672	0.3874	0.0560	0.035*	
H4B	0.8814	0.3352	−0.0342	0.035*	
N5	0.6714 (4)	0.2757 (2)	0.1666 (2)	0.0502 (9)	
H5A	0.5783	0.2280	0.1624	0.060*	
H5B	0.7700	0.2610	0.1854	0.060*	
N6	0.4605 (4)	0.2076 (2)	−0.0386 (2)	0.0442 (7)	
H6A	0.4803	0.2070	−0.0899	0.053*	
H6B	0.4238	0.1425	−0.0468	0.053*	
N7A	0.6581 (10)	−0.1254 (6)	−0.1190 (5)	0.0476 (17)	0.50
N7B	0.7260 (11)	−0.1575 (5)	−0.1417 (5)	0.0470 (18)	0.50
N8	0.8102 (4)	0.0598 (3)	0.39475 (18)	0.0438 (7)	
N9	0.6489 (8)	0.1111 (5)	0.2816 (3)	0.121 (2)	
H9A	0.6983	0.1717	0.2831	0.145*	
H9B	0.5622	0.0788	0.2358	0.145*	
N10	0.7714 (5)	−0.1240 (3)	0.2548 (2)	0.0542 (9)	
H10A	0.6636	−0.1547	0.2232	0.065*	
H10B	0.8504	−0.1594	0.2262	0.065*	
N11	1.0936 (5)	0.0862 (3)	0.3185 (3)	0.0710 (12)	
H11A	1.1563	0.0364	0.3070	0.085*	
H11B	1.1362	0.1267	0.2939	0.085*	
N12	0.6481 (4)	0.3192 (3)	0.63731 (19)	0.0453 (8)	
N13	1.1119 (4)	0.3383 (3)	0.6248 (2)	0.0517 (8)	
N14	0.7410 (4)	0.0827 (2)	0.6234 (2)	0.0461 (8)	
N15	0.8897 (4)	0.5446 (2)	0.8145 (2)	0.0436 (7)	
N16	1.0233 (4)	0.2910 (2)	0.8445 (2)	0.0420 (7)	
N17	0.5320 (4)	0.3039 (2)	0.8294 (2)	0.0447 (7)	
C1	0.9050 (6)	−0.0063 (2)	0.0941 (2)	0.0467 (10)	
C2	0.8901 (5)	0.1067 (3)	0.0100 (3)	0.0428 (9)	
C3	0.6980 (4)	0.4860 (2)	0.1070 (2)	0.0313 (7)	
H3A	0.7805	0.5103	0.1599	0.038*	
H3B	0.6382	0.5409	0.1111	0.038*	
C4	0.7947 (5)	0.4587 (2)	0.0318 (2)	0.0336 (7)	
H4C	0.7161	0.4445	−0.0201	0.040*	
H4D	0.8906	0.5151	0.0418	0.040*	
C5	0.5198 (4)	0.4091 (3)	0.1805 (2)	0.0407 (8)	
H5C	0.4037	0.3675	0.1717	0.049*	
H5D	0.5152	0.4803	0.2157	0.049*	
C6	0.6513 (5)	0.3769 (3)	0.2260 (2)	0.0462 (9)	
H6C	0.7634	0.4250	0.2432	0.055*	
H6D	0.6106	0.3754	0.2775	0.055*	
C7	0.4153 (4)	0.3767 (3)	0.0350 (2)	0.0363 (8)	
H7A	0.4504	0.3957	−0.0100	0.044*	
H7B	0.3324	0.4181	0.0638	0.044*	
C8	0.3297 (4)	0.2676 (3)	−0.0041 (3)	0.0443 (9)	
H8A	0.2798	0.2502	0.0392	0.053*	
H8B	0.2351	0.2534	−0.0501	0.053*	
C9	0.8049 (6)	−0.0906 (3)	−0.0803 (3)	0.0577 (12)	
C10	0.7119 (7)	0.1374 (4)	0.4256 (3)	0.0658 (13)	
H10C	0.7899	0.2039	0.4460	0.079*	
H10D	0.6586	0.1349	0.4741	0.079*	
C11	0.5764 (7)	0.1258 (5)	0.3590 (3)	0.0857 (18)	
H11C	0.4838	0.0672	0.3476	0.103*	
H11D	0.5252	0.1865	0.3781	0.103*	
C12	0.7226 (6)	−0.0336 (4)	0.3977 (3)	0.0624 (12)	
H12A	0.7497	−0.0275	0.4558	0.075*	
H12B	0.5961	−0.0410	0.3853	0.075*	
C13	0.7735 (6)	−0.1235 (3)	0.3375 (3)	0.0625 (12)	
H13A	0.8911	−0.1259	0.3587	0.075*	
H13B	0.6932	−0.1840	0.3326	0.075*	
C14	0.9881 (6)	0.0889 (4)	0.4435 (3)	0.0636 (12)	
H14A	0.9855	0.1309	0.5044	0.076*	
H14B	1.0294	0.0277	0.4382	0.076*	
C15	1.1079 (5)	0.1458 (3)	0.4106 (3)	0.0616 (12)	
H15A	1.0771	0.2117	0.4232	0.074*	
H15B	1.2280	0.1578	0.4381	0.074*	
C16	0.9920 (5)	0.3267 (3)	0.6547 (2)	0.0391 (8)	
C17	0.7572 (4)	0.1689 (3)	0.6537 (2)	0.0369 (8)	
C18	0.8531 (4)	0.4586 (3)	0.7763 (2)	0.0346 (7)	
C19	0.9416 (4)	0.3023 (2)	0.7939 (2)	0.0334 (7)	
C20	0.6234 (4)	0.3074 (3)	0.7823 (2)	0.0360 (7)	

### Atomic displacement parameters (Å^2^)

**Table:**

	*U*^11^	*U*^22^	*U*^33^	*U*^12^	*U*^13^	*U*^23^
Cu1	0.0546 (3)	0.0377 (2)	0.0257 (2)	−0.0003 (2)	0.0198 (2)	0.00578 (18)
Cu2	0.0308 (2)	0.0266 (2)	0.0505 (3)	0.01061 (16)	0.01054 (18)	0.02431 (19)
Fe1	0.0455 (4)	0.0175 (3)	0.0216 (3)	0.0119 (3)	0.0134 (3)	0.0098 (2)
Fe2	0.0374 (3)	0.0377 (3)	0.0257 (2)	0.0095 (2)	0.0108 (2)	0.0126 (2)
O1	0.077 (2)	0.101 (3)	0.0446 (18)	0.040 (2)	−0.0021 (16)	0.0255 (18)
N1	0.138 (4)	0.0361 (19)	0.038 (2)	−0.016 (2)	0.046 (2)	0.0044 (15)
N2	0.054 (2)	0.0361 (18)	0.115 (3)	0.0209 (16)	0.031 (2)	0.043 (2)
N3	0.0284 (13)	0.0366 (14)	0.0286 (14)	0.0148 (12)	0.0118 (11)	0.0201 (12)
N4	0.0321 (14)	0.0209 (12)	0.0325 (15)	0.0077 (11)	0.0129 (11)	0.0077 (11)
N5	0.056 (2)	0.0480 (19)	0.053 (2)	−0.0032 (16)	−0.0136 (16)	0.0363 (17)
N6	0.0465 (18)	0.0392 (17)	0.0478 (19)	0.0017 (14)	−0.0003 (15)	0.0234 (15)
N7A	0.039 (4)	0.059 (5)	0.050 (5)	−0.002 (4)	0.002 (3)	0.033 (4)
N7B	0.052 (5)	0.035 (4)	0.056 (5)	0.013 (3)	0.017 (4)	0.020 (4)
N8	0.0349 (16)	0.055 (2)	0.0249 (15)	0.0007 (14)	0.0062 (12)	0.0048 (14)
N9	0.153 (5)	0.186 (6)	0.060 (3)	0.094 (5)	0.022 (3)	0.067 (4)
N10	0.057 (2)	0.048 (2)	0.047 (2)	0.0005 (17)	0.0227 (16)	0.0110 (16)
N11	0.049 (2)	0.049 (2)	0.083 (3)	−0.0051 (17)	0.033 (2)	−0.001 (2)
N12	0.0513 (19)	0.056 (2)	0.0298 (16)	0.0171 (16)	0.0132 (14)	0.0165 (15)
N13	0.058 (2)	0.053 (2)	0.051 (2)	0.0148 (17)	0.0304 (17)	0.0238 (17)
N14	0.0515 (19)	0.0432 (19)	0.0385 (18)	0.0073 (15)	0.0203 (15)	0.0118 (15)
N15	0.0380 (16)	0.0425 (19)	0.052 (2)	0.0176 (15)	0.0094 (14)	0.0186 (16)
N16	0.0494 (18)	0.0384 (17)	0.0406 (18)	0.0113 (14)	0.0091 (15)	0.0185 (14)
N17	0.0402 (17)	0.054 (2)	0.0362 (17)	0.0093 (15)	0.0130 (14)	0.0157 (15)
C1	0.083 (3)	0.0172 (15)	0.034 (2)	0.0009 (17)	0.0239 (19)	0.0066 (14)
C2	0.049 (2)	0.0280 (17)	0.063 (3)	0.0126 (16)	0.0213 (19)	0.0265 (17)
C3	0.0400 (18)	0.0282 (16)	0.0324 (17)	0.0162 (14)	0.0139 (14)	0.0153 (14)
C4	0.0435 (19)	0.0240 (15)	0.0379 (19)	0.0098 (14)	0.0154 (15)	0.0156 (14)
C5	0.0324 (18)	0.067 (3)	0.0325 (19)	0.0157 (17)	0.0134 (14)	0.0277 (18)
C6	0.046 (2)	0.066 (3)	0.035 (2)	0.0060 (19)	−0.0012 (16)	0.032 (2)
C7	0.0353 (18)	0.053 (2)	0.0374 (19)	0.0223 (16)	0.0111 (15)	0.0303 (17)
C8	0.0282 (17)	0.069 (3)	0.049 (2)	0.0049 (17)	−0.0004 (16)	0.041 (2)
C9	0.087 (3)	0.042 (2)	0.044 (2)	−0.013 (2)	−0.008 (2)	0.031 (2)
C10	0.082 (3)	0.087 (3)	0.040 (2)	0.051 (3)	0.032 (2)	0.023 (2)
C11	0.064 (3)	0.098 (4)	0.070 (4)	0.043 (3)	0.008 (3)	0.004 (3)
C12	0.067 (3)	0.095 (4)	0.042 (2)	0.025 (3)	0.021 (2)	0.040 (3)
C13	0.069 (3)	0.056 (3)	0.048 (3)	−0.019 (2)	−0.014 (2)	0.024 (2)
C14	0.055 (3)	0.060 (3)	0.054 (3)	0.011 (2)	−0.006 (2)	0.008 (2)
C15	0.040 (2)	0.043 (2)	0.073 (3)	0.0039 (19)	0.004 (2)	0.001 (2)
C16	0.047 (2)	0.0395 (19)	0.0341 (19)	0.0125 (16)	0.0141 (16)	0.0162 (16)
C17	0.0381 (19)	0.042 (2)	0.0278 (18)	0.0068 (16)	0.0137 (14)	0.0121 (15)
C18	0.0318 (17)	0.046 (2)	0.0347 (19)	0.0168 (16)	0.0146 (14)	0.0212 (17)
C19	0.0350 (17)	0.0285 (16)	0.0340 (18)	0.0033 (14)	0.0126 (14)	0.0112 (14)
C20	0.0342 (18)	0.0410 (19)	0.0288 (17)	0.0077 (15)	0.0059 (14)	0.0112 (15)

### Geometric parameters (Å, º)

**Table:**

Cu1—N1	1.932 (3)	N9—H9B	0.9194
Cu1—N8	2.047 (3)	N9—C11	1.456 (7)
Cu1—N9	2.066 (5)	N10—H10A	0.9201
Cu1—N10	2.071 (3)	N10—H10B	0.9207
Cu1—N11	2.105 (4)	N10—C13	1.448 (5)
Cu2—N2	1.947 (3)	N11—H11A	0.9196
Cu2—N3	2.048 (2)	N11—H11B	0.9194
Cu2—N4	2.064 (3)	N11—C15	1.468 (6)
Cu2—N5	2.047 (3)	N13—C16	1.142 (4)
Cu2—N6	2.103 (3)	N14—C17	1.152 (5)
Fe1—C1	1.895 (4)	N15—C18	1.149 (4)
Fe1—C1^i^	1.895 (4)	N16—C19	1.149 (4)
Fe1—C2^i^	1.886 (3)	N17—C20	1.148 (4)
Fe1—C2	1.886 (3)	C3—H3A	0.9900
Fe1—C9	1.912 (4)	C3—H3B	0.9900
Fe1—C9^i^	1.912 (4)	C3—C4	1.511 (4)
Fe2—N12	1.657 (3)	C4—H4C	0.9900
Fe2—C16	1.941 (4)	C4—H4D	0.9900
Fe2—C17	1.934 (4)	C5—H5C	0.9900
Fe2—C18	1.941 (4)	C5—H5D	0.9900
Fe2—C19	1.938 (4)	C5—C6	1.523 (5)
Fe2—C20	1.933 (4)	C6—H6C	0.9900
O1—N12	1.141 (4)	C6—H6D	0.9900
N1—C1	1.143 (5)	C7—H7A	0.9900
N2—C2	1.148 (4)	C7—H7B	0.9900
N3—C3	1.486 (4)	C7—C8	1.498 (5)
N3—C5	1.482 (4)	C8—H8A	0.9900
N3—C7	1.484 (4)	C8—H8B	0.9900
N4—H4A	0.9201	C10—H10C	0.9900
N4—H4B	0.9194	C10—H10D	0.9900
N4—C4	1.473 (4)	C10—C11	1.464 (7)
N5—H5A	0.9190	C11—H11C	0.9900
N5—H5B	0.9201	C11—H11D	0.9900
N5—C6	1.475 (5)	C12—H12A	0.9900
N6—H6A	0.9211	C12—H12B	0.9900
N6—H6B	0.9194	C12—C13	1.456 (7)
N6—C8	1.472 (5)	C13—H13A	0.9900
N7A—C9	1.221 (8)	C13—H13B	0.9900
N7B—C9	1.165 (9)	C14—H14A	0.9900
N8—C10	1.436 (5)	C14—H14B	0.9900
N8—C12	1.484 (6)	C14—C15	1.484 (7)
N8—C14	1.496 (5)	C15—H15A	0.9900
N9—H9A	0.9191	C15—H15B	0.9900
			
N1—Cu1—N8	177.60 (14)	C13—N10—Cu1	109.2 (3)
N1—Cu1—N9	96.5 (2)	C13—N10—H10A	109.8
N1—Cu1—N10	97.96 (14)	C13—N10—H10B	110.2
N1—Cu1—N11	95.37 (18)	Cu1—N11—H11A	110.3
N8—Cu1—N9	82.80 (16)	Cu1—N11—H11B	110.1
N8—Cu1—N10	84.33 (13)	H11A—N11—H11B	108.5
N8—Cu1—N11	83.12 (14)	C15—N11—Cu1	107.9 (3)
N9—Cu1—N10	123.4 (2)	C15—N11—H11A	110.0
N9—Cu1—N11	121.7 (2)	C15—N11—H11B	110.0
N10—Cu1—N11	110.89 (16)	O1—N12—Fe2	178.1 (3)
N2—Cu2—N3	175.37 (15)	N1—C1—Fe1	176.5 (4)
N2—Cu2—N4	93.44 (12)	N2—C2—Fe1	176.1 (4)
N2—Cu2—N5	93.31 (16)	N3—C3—H3A	109.6
N2—Cu2—N6	100.50 (15)	N3—C3—H3B	109.6
N3—Cu2—N4	84.67 (10)	N3—C3—C4	110.2 (3)
N3—Cu2—N6	84.13 (11)	H3A—C3—H3B	108.1
N4—Cu2—N6	113.41 (11)	C4—C3—H3A	109.6
N5—Cu2—N3	84.58 (12)	C4—C3—H3B	109.6
N5—Cu2—N4	128.10 (12)	N4—C4—C3	108.1 (2)
N5—Cu2—N6	115.70 (13)	N4—C4—H4C	110.1
C1^i^—Fe1—C1	179.999 (1)	N4—C4—H4D	110.1
C1^i^—Fe1—C9^i^	93.61 (18)	C3—C4—H4C	110.1
C1—Fe1—C9^i^	86.39 (18)	C3—C4—H4D	110.1
C1^i^—Fe1—C9	86.39 (18)	H4C—C4—H4D	108.4
C1—Fe1—C9	93.61 (18)	N3—C5—H5C	109.7
C2—Fe1—C1^i^	89.62 (16)	N3—C5—H5D	109.7
C2^i^—Fe1—C1	89.62 (16)	N3—C5—C6	109.6 (3)
C2^i^—Fe1—C1^i^	90.38 (16)	H5C—C5—H5D	108.2
C2—Fe1—C1	90.38 (16)	C6—C5—H5C	109.7
C2^i^—Fe1—C2	180.0	C6—C5—H5D	109.7
C2—Fe1—C9	88.17 (19)	N5—C6—C5	107.7 (3)
C2^i^—Fe1—C9^i^	88.17 (19)	N5—C6—H6C	110.2
C2—Fe1—C9^i^	91.83 (19)	N5—C6—H6D	110.2
C2^i^—Fe1—C9	91.83 (19)	C5—C6—H6C	110.2
C9^i^—Fe1—C9	180.0	C5—C6—H6D	110.2
N12—Fe2—C16	97.01 (15)	H6C—C6—H6D	108.5
N12—Fe2—C17	94.65 (16)	N3—C7—H7A	109.5
N12—Fe2—C18	94.85 (15)	N3—C7—H7B	109.5
N12—Fe2—C19	175.76 (15)	N3—C7—C8	110.7 (3)
N12—Fe2—C20	94.57 (15)	H7A—C7—H7B	108.1
C17—Fe2—C16	91.02 (14)	C8—C7—H7A	109.5
C17—Fe2—C18	170.45 (15)	C8—C7—H7B	109.5
C17—Fe2—C19	83.43 (14)	N6—C8—C7	108.2 (3)
C18—Fe2—C16	86.88 (14)	N6—C8—H8A	110.0
C19—Fe2—C16	86.82 (14)	N6—C8—H8B	110.0
C19—Fe2—C18	87.15 (14)	C7—C8—H8A	110.0
C20—Fe2—C16	168.06 (15)	C7—C8—H8B	110.0
C20—Fe2—C17	90.94 (14)	H8A—C8—H8B	108.4
C20—Fe2—C18	89.25 (14)	N7A—C9—Fe1	161.2 (6)
C20—Fe2—C19	81.71 (14)	N7B—C9—Fe1	159.4 (6)
C1—N1—Cu1	163.4 (4)	N8—C10—H10C	109.4
C2—N2—Cu2	165.0 (4)	N8—C10—H10D	109.4
C3—N3—Cu2	106.62 (17)	N8—C10—C11	111.2 (4)
C5—N3—Cu2	108.6 (2)	H10C—C10—H10D	108.0
C5—N3—C3	111.1 (3)	C11—C10—H10C	109.4
C5—N3—C7	111.1 (2)	C11—C10—H10D	109.4
C7—N3—Cu2	107.8 (2)	N9—C11—C10	109.9 (4)
C7—N3—C3	111.4 (2)	N9—C11—H11C	109.7
Cu2—N4—H4A	110.1	N9—C11—H11D	109.7
Cu2—N4—H4B	109.8	C10—C11—H11C	109.7
H4A—N4—H4B	108.3	C10—C11—H11D	109.7
C4—N4—Cu2	108.82 (19)	H11C—C11—H11D	108.2
C4—N4—H4A	109.9	N8—C12—H12A	109.0
C4—N4—H4B	109.8	N8—C12—H12B	109.0
Cu2—N5—H5A	110.1	H12A—C12—H12B	107.8
Cu2—N5—H5B	110.2	C13—C12—N8	113.0 (4)
H5A—N5—H5B	108.6	C13—C12—H12A	109.0
C6—N5—Cu2	107.3 (2)	C13—C12—H12B	109.0
C6—N5—H5A	110.4	N10—C13—C12	110.8 (4)
C6—N5—H5B	110.2	N10—C13—H13A	109.5
Cu2—N6—H6A	110.3	N10—C13—H13B	109.5
Cu2—N6—H6B	110.4	C12—C13—H13A	109.5
H6A—N6—H6B	108.6	C12—C13—H13B	109.5
C8—N6—Cu2	106.8 (2)	H13A—C13—H13B	108.1
C8—N6—H6A	110.3	N8—C14—H14A	109.6
C8—N6—H6B	110.4	N8—C14—H14B	109.6
C10—N8—Cu1	109.5 (3)	H14A—C14—H14B	108.1
C10—N8—C12	111.4 (3)	C15—C14—N8	110.1 (4)
C10—N8—C14	113.5 (3)	C15—C14—H14A	109.6
C12—N8—Cu1	106.4 (2)	C15—C14—H14B	109.6
C12—N8—C14	107.3 (3)	N11—C15—C14	108.2 (4)
C14—N8—Cu1	108.5 (3)	N11—C15—H15A	110.1
Cu1—N9—H9A	110.5	N11—C15—H15B	110.1
Cu1—N9—H9B	109.6	C14—C15—H15A	110.1
H9A—N9—H9B	108.4	C14—C15—H15B	110.1
C11—N9—Cu1	108.1 (3)	H15A—C15—H15B	108.4
C11—N9—H9A	111.0	N13—C16—Fe2	177.2 (3)
C11—N9—H9B	109.1	N14—C17—Fe2	177.7 (3)
Cu1—N10—H10A	109.5	N15—C18—Fe2	178.3 (3)
Cu1—N10—H10B	109.9	N16—C19—Fe2	175.7 (3)
H10A—N10—H10B	108.3	N17—C20—Fe2	176.2 (3)
			
Cu1—N8—C10—C11	34.4 (5)	N8—Cu1—N10—C13	−8.5 (3)
Cu1—N8—C12—C13	36.7 (4)	N8—Cu1—N11—C15	−16.3 (3)
Cu1—N8—C14—C15	37.6 (4)	N8—C10—C11—N9	−48.8 (7)
Cu1—N9—C11—C10	37.7 (6)	N8—C12—C13—N10	−46.3 (5)
Cu1—N10—C13—C12	30.9 (4)	N8—C14—C15—N11	−52.9 (5)
Cu1—N11—C15—C14	40.9 (4)	N9—Cu1—N1—C1	101.8 (15)
Cu2—N3—C3—C4	41.0 (3)	N9—Cu1—N8—C10	−10.3 (4)
Cu2—N3—C5—C6	32.9 (4)	N9—Cu1—N8—C12	110.2 (3)
Cu2—N3—C7—C8	37.1 (3)	N9—Cu1—N8—C14	−134.6 (3)
Cu2—N4—C4—C3	36.1 (3)	N9—Cu1—N10—C13	−86.0 (3)
Cu2—N5—C6—C5	43.9 (3)	N9—Cu1—N11—C15	60.8 (4)
Cu2—N6—C8—C7	41.1 (3)	N10—Cu1—N1—C1	−133.0 (15)
N1—Cu1—N9—C11	167.2 (5)	N10—Cu1—N8—C10	−135.1 (3)
N1—Cu1—N10—C13	170.8 (3)	N10—Cu1—N8—C12	−14.6 (3)
N1—Cu1—N11—C15	161.8 (3)	N10—Cu1—N8—C14	100.6 (3)
N2—Cu2—N4—C4	173.0 (2)	N10—Cu1—N9—C11	63.2 (5)
N2—Cu2—N5—C6	154.9 (3)	N10—Cu1—N11—C15	−97.6 (3)
N2—Cu2—N6—C8	163.0 (2)	N11—Cu1—N1—C1	−21.0 (15)
N3—Cu2—N4—C4	−11.3 (2)	N11—Cu1—N8—C10	113.0 (3)
N3—Cu2—N5—C6	−20.9 (2)	N11—Cu1—N8—C12	−126.5 (3)
N3—Cu2—N6—C8	−17.0 (2)	N11—Cu1—N8—C14	−11.3 (3)
N3—C3—C4—N4	−52.2 (3)	N11—Cu1—N9—C11	−92.4 (5)
N3—C5—C6—N5	−51.7 (4)	N11—Cu1—N10—C13	71.9 (3)
N3—C7—C8—N6	−53.4 (4)	C1^i^—Fe1—C9—N7A	−113.6 (14)
N4—Cu2—N2—C2	121.7 (13)	C1—Fe1—C9—N7A	66.4 (14)
N4—Cu2—N3—C3	−16.2 (2)	C1—Fe1—C9—N7B	−134.4 (13)
N4—Cu2—N3—C5	−136.0 (2)	C1^i^—Fe1—C9—N7B	45.6 (13)
N4—Cu2—N3—C7	103.6 (2)	C2—Fe1—C9—N7A	−23.9 (14)
N4—Cu2—N5—C6	57.9 (3)	C2^i^—Fe1—C9—N7A	156.1 (14)
N4—Cu2—N6—C8	−98.5 (2)	C2^i^—Fe1—C9—N7B	−44.6 (13)
N5—Cu2—N2—C2	−6.9 (13)	C2—Fe1—C9—N7B	135.4 (13)
N5—Cu2—N3—C3	113.0 (2)	C3—N3—C5—C6	−84.1 (3)
N5—Cu2—N3—C5	−6.8 (2)	C3—N3—C7—C8	153.8 (3)
N5—Cu2—N3—C7	−127.3 (2)	C5—N3—C3—C4	159.2 (3)
N5—Cu2—N4—C4	−90.1 (2)	C5—N3—C7—C8	−81.8 (3)
N5—Cu2—N6—C8	64.1 (2)	C7—N3—C3—C4	−76.4 (3)
N6—Cu2—N2—C2	−123.8 (13)	C7—N3—C5—C6	151.3 (3)
N6—Cu2—N3—C3	−130.4 (2)	C10—N8—C12—C13	156.0 (4)
N6—Cu2—N3—C5	109.8 (2)	C10—N8—C14—C15	−84.3 (4)
N6—Cu2—N3—C7	−10.7 (2)	C12—N8—C10—C11	−83.0 (5)
N6—Cu2—N4—C4	70.0 (2)	C12—N8—C14—C15	152.2 (4)
N6—Cu2—N5—C6	−101.8 (2)	C14—N8—C10—C11	155.7 (5)
N8—Cu1—N9—C11	−15.1 (4)	C14—N8—C12—C13	−79.2 (4)

Symmetry code: (i) −*x*+2, −*y*, −*z*.

### Hydrogen-bond geometry (Å, º)

**Table:**

*D*—H···*A*	*D*—H	H···*A*	*D*···*A*	*D*—H···*A*
N4—H4*A*···N15^ii^	0.92	2.20	3.020 (4)	149
N4—H4*B*···N16^iii^	0.92	2.37	3.248 (4)	159
N5—H5*A*···N7*A*^iv^	0.92	2.09	2.979 (9)	162
N5—H5*A*···N7*B*^iv^	0.92	2.41	3.267 (9)	154
N6—H6*A*···N17^iii^	0.92	2.43	3.242 (5)	147
N10—H10*A*···N17^v^	0.92	2.29	3.060 (5)	141
N10—H10*B*···N16^vi^	0.92	2.27	3.151 (5)	160

Symmetry codes: (ii) −*x*+2, −*y*+1, −*z*+1; (iii) *x*, *y*, *z*−1; (iv) −*x*+1, −*y*, −*z*; (v) −*x*+1, −*y*, −*z*+1; (vi) −*x*+2, −*y*, −*z*+1.
